# The double-edged sword effect of algorithmic management on work engagement of platform workers: the roles of appraisals and resources

**DOI:** 10.3389/fpsyg.2025.1522088

**Published:** 2025-06-25

**Authors:** Feifei Li, Xiaohui Zhan, Yun Liu

**Affiliations:** ^1^School of Management, Nanjing University of Posts and Telecommunications, Nanjing, China; ^2^School of International Business, Jinan University, Zhuhai, China; ^3^Department of Marketing and Logistics Management, Nanjing University of Finance and Economics, Nanjing, China

**Keywords:** algorithmic management, challenge appraisal, threat appraisal, emotional stability, perceived organizational support, work engagement

## Abstract

**Purpose:**

This study aims to explore how food delivery workers' perceptions of threat and challenge mediate the influence of algorithmic management on their work engagement, and to examine the moderating roles of emotional stability and perceived organizational support (POS) in this process.

**Design/methodology/approach:**

The present research conceptualized and validated a framework to investigate the double-edged sword influence of algorithmic management on work engagement, highlighting its potential to be both beneficial and detrimental. Drawing from a sample of 292 delivery workers working in two major food delivery platforms in China, this study employed SPSS 20.0 and MPLUS 7.4 for the statistical examination of the hypotheses.

**Findings:**

The findings reveal that threat appraisal serves as a negative mediator between algorithmic management and work engagement, while challenge appraisal served as a positive mediator in this relationship. Emotional stability and perceived organizational support (POS) acted as moderators of these effects, indicating that when algorithmic management was perceived more as a challenge than a threat, it was more likely to enhance the engagement behavior at work.

**Practical implications:**

This study uncovers algorithmic management's dual effects. Employees with high emotional stability and organizational support (POS) who view it as a challenge (not threat) show stronger work engagement. Managers should cultivate these psychological resources to enhance technology adoption success.

**Originality/value:**

This study advances the algorithmic management literature by investigating its dual effects on food delivery workers' work engagement. We uncover the underlying mediating mechanisms and demonstrate how emotional stability and perceived organizational support (POS) moderate these relationships.

## 1 Introduction

With the development of new-generation digital technologies, the use of algorithms for management has become increasingly prevalent in the gig economy (Kellogg et al., 2020), covering a wide range of industries from car-hailing services (such as Uber and Lyft), food delivery services (such as Meituan and Amazon Flex), to logistics, retail, healthcare, and more (Stark and Pais, 2021; Möhlmann et al., 2021; Liu et al., 2025; Parent-Rocheleau and Parker, 2022). Among them, food delivery platforms, as typical users of algorithmic management, take algorithmic technology as the core to assign food-delivery orders to workers, track their locations, appraise performance, and even implement rewards and punishments sometimes (Li et al., 2024; Rosenblat and Stark, 2016). In doing so, food delivery platforms are able to enhance operational efficiency and financial performance (Jarrahi et al., 2021).

As a supplement and substitute for traditional labor management methods (Parent-Rocheleau and Parker, 2022), algorithmic management requires minimal human intervention while enabling online food delivery platforms to employ and utilize a large number of delivery workers at a relatively low cost (Schmidt, 2017). These delivery workers form the most important part of the online food delivery service network (Sun, 2019); hence the platforms need to continuously improve their work engagement. However, compared with employees in traditional companies, these delivery workers are more likely to exhibit lower work engagement and higher turnover rates due to the flexibility and instability of the gig economy (Lin et al., 2020; Li et al., 2024). According to the report from the Meituan Research Institute, in 2023, among the food delivery workers who earned income by taking orders, 11% were high-frequency order takers (those who took orders for more than 260 days throughout the year), 41% were low-frequency order takers (those who took orders for 30 to 260 days throughout the year), and 48% were casual order takers (those who took orders for no more than 30 days throughout the year). Another survey conducted by the China New Employment Forms Research Center also shows that in 2024, the turnover rate in the food delivery industry is generally between 30 and 40%. Obviously, increasing the work engagement of delivery workers is very important for platforms to reduce costs, improve service quality, and enhance organizational efficiency. Therefore, ensuring the work engagement of food delivery workers has become an urgent issue for online food delivery platforms.

Given the widespread application and significance of algorithmic management across different industries worldwide, scholars have increasingly paid attention to this topic and explore its impact on employees' work-related states. For example, based on the Job Demands-Resources model, Parent-Rocheleau and Parker (2022) have explored how algorithmic management can affect the motivation and well-being of workers through two pathways: job resources (e.g., job autonomy, job complexity) and job demands (e.g., workload, physical demands). Wang et al. (2022), drawing on the Conservation of Resources theory, argued that gig workers' psychological resources, such as affective commitment and trust, play mediating roles in the relationship between algorithmic management and work engagement. Lang et al. (2023) have proposed that gig workers' emotional state, namely burnout, mediate the relationship between algorithmic management and their work engagement.

Although these studies may have revealed the mechanisms by which algorithmic management affects workers' work engagement, they have largely ignored the significant role of gig workers' cognition of algorithmic management in this process (Li et al., 2024; Zhang et al., 2023). As a management approach centered on algorithmic technology, algorithmic management can not only control the work outcomes of food delivery workers by setting precise and rigid assessment standards, but also regulate their work behaviors by establishing standardized work processes to ensure that employees follow established rules and standards when performing tasks. Therefore, it can be considered a unique source of work stress. In light of this situation, we believe that the cognitive appraisal of food delivery workers is likely to play a significant role in the relationship between algorithmic management and work-related outcomes. In summary, we aim to link algorithmic management to the work engagement of food delivery workers and explore its underlying mechanisms from the perspective of stress appraisal.

As a form of labor control that embedded in almost every aspect of food delivery work (Li et al., 2024), algorithmic management may have double-edged sword effects on workers, potentially increasing or decreasing their work engagement. On the one hand, the change from human leader to algorithmic technology can improve the work efficiency of food delivery workers through functions such as optimizing delivery routes, intelligent task allocation, and real-time navigation support (Pei et al., 2021; Wood et al., 2019). Algorithm technology can also dynamically adjust delivery fees and rewards based on market demand and order dynamics, thereby increasing the income of food delivery workers (Kellogg et al., 2020). Hence, food delivery workers may increase their work engagement due to these potential benefits. On the other hand, algorithmic management force food delivery workers to complete orders within a short period of time (Wood et al., 2019), which may lead to a significant sense of pressure for delivery workers (Cram et al., 2022), especially when traffic conditions are poor. Meanwhile, the algorithmic system constantly monitors the location, speed, and behavior of delivery workers, which may make them feel a lack of autonomy and control (Kellogg et al., 2020). In addition, algorithmic management reduces social interactions between delivery workers and their colleagues, which may lead to feelings of loneliness and isolation (Lei, 2021). In this regard, delivery workers may reduce their work engagement because of these potential damages.

Based on the above analysis, our study thus proposes a dual-path model to clarify the impact of algorithmic management on the work engagement of food delivery workers, on the basis of transactional theory of stress (Lazarus and Folkman, 1984). According to this theory, when facing external stressors or stressful situations relevant to themselves, individuals often make differentiated cognitive appraisals based on their own feelings and the characteristics of these situations, and then adopt different coping strategies. Algorithmic management can be seen as a potential stressor closely related to food delivery workers, as it comprehensively and profoundly affects the content, style, and environment in which workers complete their tasks, bringing them an entirely new work experience (Pei et al., 2021). In line with this logic, food delivery workers may conduct challenge appraisals or threat appraisals of algorithmic management, and subsequently react in different ways. To be specific, we propose that food delivery workers who develop a threat appraisal toward algorithmic management may expect it as providing informational support for task completion, thereby increasing work engagement. Conversely, a challenge appraisal of algorithmic management is more likely to lead workers to view it as a constraint, resulting in decreased work engagement.

Additionally, it is important to investigate the boundary conditions that influence the extent to which employees conduct challenge and hindrance appraisals of algorithmic management. The transactional theory of stress proposed by Lazarus and Folkman (1984) further indicates that an individual's cognitive appraisal of stressors is influenced not only by the nature of the work stressor itself but also by the resources available to the individual. These resources, whether related to the individual or to the environment in which they are situated, facilitate effective coping with work stress (Bakker and Demerouti, 2017) and regulate their attention to certain features (i.e., positive or negative aspects) of the stressor (Liu et al., 2022). Expanding upon Lazarus' (1966) transactional theory of stress and in response to the appeal issued by Li et al. (2024), we propose that delivery works' appraisal of algorithmic management may vary depending on individual resources (emotional stability) and job resource (POS). We choose emotional stability as an important individual resources that moderates the relationship between algorithmic management and challenge/threat appraisal, because emotional stability refers to the trait that enables individuals to effectively adapt to and cope with stress, loss, hardship, or adversity (Alessandri et al., 2018; David et al., 2020). Different from other individual factors, emotional stability is considered the most important personality trait in the workplace after conscientiousness (Kundi et al., 2022) and represents an individual's ability in handling negative emotions effectively, encompassing stress, anxiety, and anger (Alessandri et al., 2018). Moreover, some previous studies have demonstrated can influence the process by which individuals cope with stressors, primarily by helping them to deal with stress in a positive manner rather than focusing on the negative aspects of the stressors (Kundi et al., 2022; Kaiser and Ozer, 1997). Considering the fact that algorithmic management may trigger negative emotions (Lee, 2018), we posit that in exploring the relationship between algorithmic management and cognitive appraisals, emotional stability may act as an important individual resource.

Furthermore, we examine POS as an important job resource that can regulate the relationship between algorithmic management and challenge/threat appraisal, because it reflects, the overall possibility for individuals to gain access to important items, energy, and social resources provided by their work colleagues during their job (Sarwar et al., 2020). POS refers to the degree to which employees feel their efforts are recognized and appreciated by the organization, as well as the extent to which the firm prioritizes their wellbeing (Eisenberger et al., 1986). Previous research has demonstrated that POS can mitigate the detrimental impacts of stressors on various work outcomes by facilitating employees' stress management (Stamper and Johlke, 2003; Sarwar et al., 2020). Therefore, in this study, we suggest that POS can provide the resources that delivery workers need to pay attention to the positive aspects of algorithmic management.

Accordingly, the current study benefits extant literature in several ways. First, taking algorithmic management as a whole, we explore the double-edged sword effect of algorithmic management on the attitudes and behaviors of food delivery workers. Previous studies have not yet reached a consensus on the impact of algorithmic management on employees' work attitudes and behaviors (Benlian et al., 2022; Duggan et al., 2020). Some scholars believe that algorithmic management has a positive impact on employees, such as improving job autonomy (Wood et al., 2019), positive moods (Kellogg et al., 2020), and high performance (Pei et al., 2021). While other scholars hold the opposite view, arguing that algorithmic management could have negative impacts on employees, leading to problems such as social isolation (Lei, 2021), overwork (Wood et al., 2019), job insecurity (Kellogg et al., 2020), and wellbeing (Cram et al., 2022). However, limited empirical studies have focused on the double-edged sword effect of algorithmic management on workers (Pei et al., 2021; Lang et al., 2023; Li et al., 2024). Building on such work, our study is the first empirical study to explore the double-sword effect of algorithmic management on the work engagement of food delivery workers, thus enriching the nomological network of algorithmic management consequences.

Second, we identified two distinctive types of stress appraisals (challenge and threat) as mediating variables to explain how algorithmic management promotes or hinders the work engagement of food delivery workers. Among the few studies that focusing on the consequences of algorithmic management, scholars have primarily explored the mediating roles of work condition change and workers' affective response (Parent-Rocheleau and Parker, 2022; Wang et al., 2022), with less attention paid to the role of their cognitive process (Li et al., 2024; Lang et al., 2023). Our study, by considering challenge and threat appraisals as mediating variables, can help explain the double-edged sword effect of algorithmic management on the work engagement of food delivery workers, thereby shedding light on the scholarly understanding of why algorithmic management may promote or hinder work engagement of food delivery workers.

Third, we identify POS and emotional stability as two key boundary conditions. Among the few studies, previous studies have mainly examined the moderating roles of regulatory focus (promotion and prevention, Lang et al., 2023), platform algorithmic fairness (Wang et al., 2022), and employment type (part-time and full-time, Pei et al., 2021) on the relationship between algorithmic management and delivery workers' attitudes and behaviors. Despite these fruitful studies, further examinations of other important individual and contextual characteristics are warranted to analyze the contingencies of algorithmic management' s consequences on workers. Thus, our study advances the literature on algorithmic management by introducing POS and emotional stability to explore the contingencies for the relationship between algorithmic management and work engagement of food delivery workers.

## 2 Theoretical development and hypotheses

### 2.1 Algorithmic management, threat appraisal and work engagement

Compared to other theories consider a stressor in isolation as an inhibitor or facilitator, the transactional theory of stress (TTS) posits that the different consequences of a stressor originate from employees' divergent responses based on how they appraise the stressor (LePine, 2005). More specifically, TTS emphasizes that external factors in essence are not direct triggers causing an individual's reaction, but it is his/her transactional, interactive and dynamic cognitive process with external factors determining whether the stressor produces a positive or negative effect. Meanwhile, they tend to appraise environmental conditions (stressors) as hindrance when such stressors exceed their ability to cope, thus (a) interfering with and thwarting the process of goal attainment, and (b) evoking negative emotions (Lazarus and Folkman, 1984). TTS also argues that the primary appraisals, in turn, influence individuals' work attitudes, satisfaction, and performance (Dewe, 2013; Webster et al., 2010; Zhang et al., 2015). Taken together, TTS indicates that it is an individual's primary appraisal, rather than environmental conditions (stressors), that directly determine his/her psychological and behavioral reactions.

The concept of threat appraisal pertains to individuals perceiving stressors as threats to their wellbeing, impeding and obstructing their pursuit of personal goals and growth (Lazarus and Folkman, 1984). Work engagement is characterized as “a state of positive vigor, dedication, and deep absorption in one's work” (Schaufeli et al., 2002, pp. 74).

Following the TTS, we argue that appraisals can function as mechanisms that underline the association between AM and work engagement. To be more specific, we propose that AM can be appraised as threat stress in the following three ways. Firstly, the AM reduce the work autonomy of the deliveryman and make them “puppet” of the organization. For example, Duggan et al. (2023) found that the algorithmic HRM system acts as a pervasive and defining control mechanism in task allocation, performance management, reward distribution, and aligning employee actions with organizational goals. Customers can upload real-time information about workers' performance, and the algorithmic system promptly identifies and notifies workers to adjust their behavior or attitude accordingly (Green, 2018). Matherne and O'Toole (2017) also noted that Uber frequently uses algorithmic system to send information to drivers, reminding them to strive to improve their service attitude and work quality in order to control drivers' misconduct and uncivil behavior. And it is conceivable that Uber drivers are frequently guided by the algorithmic system in managing customer conflicts and maintaining customer relations during their work (Matherne and O'Toole, 2017). Thus, AM leads to delivery workers being unable to autonomously decide on their work way (Duggan et al., 2023), thereby reducing their level of work engagement. Secondly, the electronic monitoring systems of AM can infringe on workers' privacy. For example, algorithms can track various information about workers, including their location and speed (Norlander et al., 2021), tracking delivery workers' service attitude, work progress, and other metrics can enhance dissatisfaction with the algorithm system and even harm workers' health (Bhave et al., 2020; Harris et al., 2022; Kulkarni, 2020), thereby leading to a decrease in work engagement. Thirdly, AM imposes strict evaluation systems on delivery workers. For instance, being rated poorly or receiving complaints can lead to fines or even account suspension, which can evoke resistance among delivery workers and impact their work engagement. Reputation significantly impacts job opportunities and salary levels, yet platforms expose workers to the uncertain risk of customer evaluations (Wei and Thomas MacDonald, 2022). Without explanation, platforms often penalize work that dissatisfies customers, leading to feelings of unfair treatment among delivery workers (Fieseler et al., 2019). Therefore, we posit the following:

**Hypothesis 1**. Threat appraisal negatively mediates the relationship between AM and work engagement.

### 2.2 Algorithmic management, challenge appraisal and work engagement

The challenge appraisal refers to individuals' perception of stressors as job demands conductive to one's growth and development in gaining and mastering professional knowledge (Lazarus and Folkman, 1984). According to TTS, individuals tend to view environmental conditions (stressors) as challenge stress when they perceive it as offering personal growth, mastery, and potential rewards. Thus, we propose that delivery workers can also appraise AM as challenge stress for the following reasons. Firstly, as a technological partner, AM provides information support and assistance to delivery employees work (Pei et al., 2021). This can lead to enhancements in their productivity and performance, such as maintaining continuous focus and adhering to platform rules. Secondly, AM implements reward and punishment mechanisms, such as order rush rewards and full attendance rewards (Ravid et al., 2020). These challenging appraisals can stimulate delivery workers' job motivation, enhance their enthusiasm for work, and ultimately increase their work commitment. Moreover, in line with TTS, individuals' assessment of stress has the potential to impact their work performance (Lazarus and Folkman, 1984). When delivery workers perceive AM as a tool that enhances their personal growth and wellbeing, they are inclined to dedicate more time and effort to the platform (Rosenblat and Stark, 2016). They may also demonstrate a higher level of enthusiasm and commitment. Thus, we propose:

**Hypothesis 2**. Challenge appraisal positively mediates the relationship between AM and work engagement.

### 2.3 The impact of resources on the stress process

The transactional stress theory (Lazarus and Folkman, 1984) further proposes that an individual's cognitive appraisal of a stressor depends not only on the nature of the stressor but also on the resources available to the individual to cope with the stressor (Liu et al., 2022). These resources, which come from the individual or the social environment in which the individual is situated, can help individuals effectively deal with stressors (Bakker and Demerouti, 2017). Therefore, we examine how personal resources (emotional stability) and work resources (perceived organizational support) influence the relationship between algorithmic management and different types of appraisals.

#### 2.3.1 The Moderating role of emotional stability

We propose that, as a personal resource, the emotional stability of food delivery drivers will influence the extent to which algorithmic management triggers different appraisals. According to transactional stress theory (Lazarus and Folkman, 1984), certain individual traits can affect the stress process by altering the way individuals process stress information (Fugate et al., 2008). As a stable individual trait, emotional stability reflects the degree of emotional fluctuation when facing external or internal environmental factors (Donnellan et al., 2006), essentially indicating an individual's emotional susceptibility or sensitivity (Parker et al., 2021). It can influence the individual's appraisals of algorithmic management because individuals with high emotional stability are more likely to focus on the positive aspects contained in algorithmic management and find resources to effectively cope with the pressure of algorithmic management.

Previous research has indicated that emotional stability is negatively correlated with some stress-related outcomes, such as job burnout (Alessandri et al., 2018; Alarcon et al., 2009), emotional exhaustion (David et al., 2020; Liu and Yu, 2019), anxiety (Ho et al., 2013), and anger (Bujor and Turliuc, 2020; Czarna et al., 2021). Emotional stability captures an individual's tendency to effectively cope with stress, loss, hardship, or adversity (Alessandri et al., 2018; David et al., 2020), helping individuals to remain calm in novel and stressful situations (Rogers and Barber, 2019). More importantly, previous studies have found that emotional stability plays the most central role in shaping an individual's reactivity to stressors (Alessandri et al., 2018). Individuals with high emotional stability can remain calm in stressful situations and are more likely to find effective strategies to cope with stressors, which provides a basis for their assessment of algorithmic management, making them less likely to appraise algorithmic management as a threat (Rogers and Barber, 2019; Kundi et al., 2022; Gallagher, 1990). This suggests that by highlighting the potential benefits and opportunities involved in algorithmic management and encouraging individuals to actively seek coping resources, emotional stability strengthens the positive relationship between algorithmic management and challenge appraisals and mitigates the positive relationship between algorithmic management and threat appraisals. Therefore, we propose the following hypothesis:

**Hypothesis 3a**. Emotional stability moderates the positive relationship between algorithmic management and threat appraisal, resulting in a weaker relationship among employees with high emotional stability.
**Hypothesis 3b**. Emotional stability moderates the positive relationship between algorithmic management and challenge appraisal, resulting in a stronger relationship among employees with high emotional stability.

#### 2.3.2 The moderating role of perceived organization support (POS)

We argue that as a job resource, POS will influence how delivery workers appraisal and respond to algorithmic management. In particular, in stressful situations, employees who can obtain high levels of social and emotional support from the platform organization will tend to make challenging appraisals of algorithmic management and reduce their threatening appraisals of stress. POS represents an individual's subjective assessment of an organization, indicating the degree to which they believe the organization values their contributions and prioritizes their overall welfare (Eisenberger et al., 2001). It is viewed as a crucial determinant of both job satisfaction and work effectiveness (Eisenberger et al., 2001). Studies indicate that employees who perceive higher levels of organizational support are inclined to develop a stronger sense of commitment to the organization and exhibit a more positive attitude toward its tasks (Eisenberger et al., 2001). Therefore, we believe that POS is well suited to amplify the positive appraisal of algorithmic management.

High levels of POS imply that the organization values workers' professional growth and contributions, prioritizes their wellbeing (Eisenberger et al., 2001), and helps them cope with the emotional pain brought about by stressors by providing emotional resources (Sarwar et al., 2020). Specifically, by offering food delivery workers support related to career development, recognizing and valuing their contributions and wellbeing, POS may influence stress appraisals as challenges or threats. For one thing, the support and resources represented by POS will enhance individuals' confidence in facing stressors (Venkatachalam, 1995) and prompt them to actively seek effective coping strategies, allowing them to recognize the positive aspects of stressors. For another, high POS means that employees can obtain more emotional comfort from the workplace, such as receiving more sympathy and consolation from leaders or colleagues when encountering negative experiences due to stressors (Eisenberger et al., 2001), which can greatly alleviate the emotional pain caused by stress (Sarwar et al., 2020) and encourage individuals to make positive evaluations of stressors. Through these processes, they can alleviate employees' stress experience when facing algorithmic management, leading them to perceive its positive aspect. Therefore, workers in the case of high (rather than low) POS are more likely to appraise algorithmic management as a challenge (i.e., an accentuating interaction effect). Instead, they are less likely to appraise it as a threat (i.e., a mitigating interaction effect). Thus, we propose the following research hypothesis:

**Hypothesis 4a**. POS attenuates the positive correlation between AM and threat appraisal, resulting in a weaker relationship among employees with high POS.
**Hypothesis 4b**. POS enhances the positive correlation between AM and challenge appraisal, strengthening this relationship among employees with high POS.

### 2.4 A moderated mediation model

**Figure 1:**
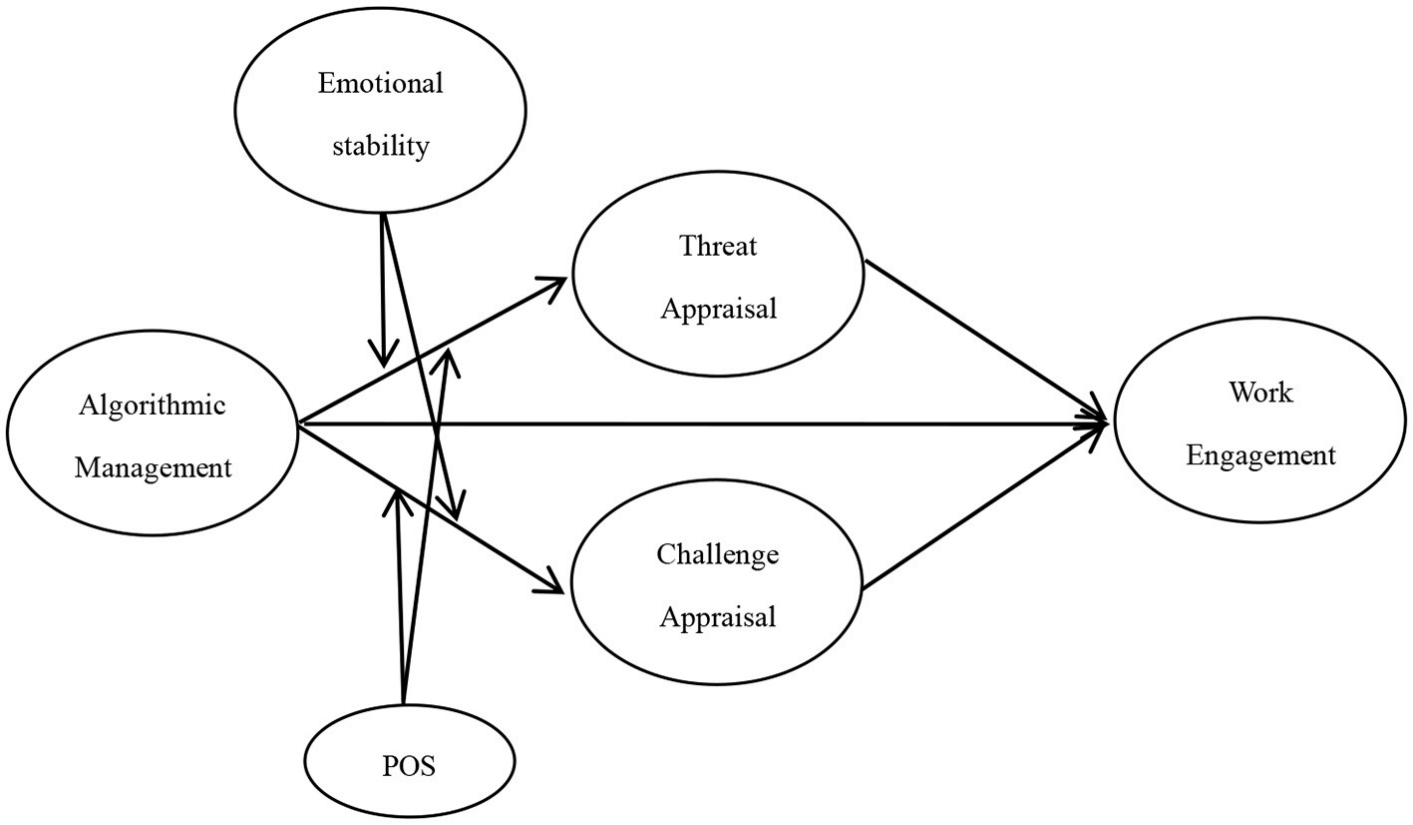
Theoretical framework.

As previously mentioned, we hypothesize that emotional stability and POS will moderate the differential effects of algorithmic management on stress appraisals, which in turn will affect the work engagement of workers. Specifically, individuals with high (vs. low) emotional stability will have more personal resources that can help them to remain calm in the face of pressure and focus on the positive aspects of algorithmic management, making them less likely to perceive algorithmic management as a threat. By reducing the threatening appraisal of algorithmic management, the damage to employee work engagement will be minimized. Furthermore, a high (vs. low) POS indicates that employees can draw more career development and emotional resources from their work environment, which will encourage them to be more likely to view algorithmic management as a challenge rather than a threat. By focusing on the positive aspects of algorithmic management, employee work engagement will be enhanced. In summary, we propose the model in Figure 1, where algorithmic management is positively related to challenge and threat appraisals, which subsequently relate to workers work engagement. Additionally, the indirect effects are moderated by emotional stability and POS. Therefore, we propose the following hypotheses:

**Hypothesis 5a**. The indirect impact of AM on work engagement through threat appraisal will be stronger when emotional stability is lower rather than higher.
**Hypothesis 5b**. The indirect impact of AM on work engagement through challenge appraisal will be stronger when emotional stability is higher rather than lower.
**Hypothesis 6a**. The indirect influence of AM on work engagement via threat appraisal will be more robust when POS is lower compared to higher.
**Hypothesis 6b**. The indirect influence of AM on work engagement via challenge appraisal will be stronger with higher POS than with lower POS.

## 3 Methods

### 3.1 Sample selection and procedure

Data were collected from food delivery workers employed by two major food delivery platforms in China (i.e., Eleme and Meituan). To obtain as many samples as possible, two main data collection methods are adopted: (1) one of the authors personally registered on the delivery platform, experienced the delivery work as a delivery rider, developed relationships with some delivery workers and distributed questionnaires to them; (2) one of the authors observed outside some food delivery stations and distribute questionnaires to food delivery workers. After data collection, we tested the sample collected by the two different methods, and the results showed no significant difference. Before filling out the questionnaire, we informed the participants about the survey's primary objective and assured them of the confidentiality of the information provided.

In addition, to reduce common method bias, a three-stage research approach was implemented, as recommended by Podsakoff et al. (2012). Specifically, in the first stage, participants completed a survey that encompassed demographic variables, algorithmic management, emotional stability and POS. Two weeks later, in the second stage, the questionnaire measure demographic variables and mediating variables, including threat and challenge appraisals. During the third stage, another 2 weeks later, the participants were asked to complete work engagement scales. Using demographic information (i.e., date of birth, sum of height, and Chinese zodiac signs) in three waves, we ensured that all three phases were conducted with the same participants. Initially, 532 delivery workers completed the survey in the first wave. In the second wave, 421 participants completed the survey and then we received 292 surveys in the third wave. The final sample includes 38 women (13%) and 254 men (87%), with ages varying from 19 to 46 (M = 24.3 years, SD = 1.025). The average tenure for the participants was 1.37 years (SD = 2.100). Of the respondents, 10.7% were junior high school graduates, 75.2% were high school graduates, and 14.1% completed technical college or above.

### 3.2 Measures

Participants were requested to rate each item on a 5-point Likert scale, with 1 representing strong disagreement and 5 representing strong agreement.

#### 3.2.1 Algorithmic management

Algorithmic management was measured with a 11-item scale developed by Pei et al. (2021). Sample items are “I feel that the algorithm system of the food delivery platform provides me with a large amount of information support related to completing work” and “I feel that the algorithm system of the food delivery platform will continue to follow up on my work progress” (α = 0.910).

#### 3.2.2 Threat appraisal

Threat appraisal was assessed using four items borrowed from Drach-Zahavy and Erez (2002). Sample items are “The algorithm system seems like a threat to me” and “I'm worried that the algorithm system might reveal my weaknesses” (α = 0.806).

#### 3.2.3 Challenge appraisal

We measured challenge appraisal using four items from Drach-Zahavy and Erez (2002). Sample items are “The algorithm system seems like a challenge to me” and “The algorithm system provides opportunities to overcome obstacles” (α = 0.804).

#### 3.2.4 Work engagement

Work engagement was measured with a 5-item scale borrowed from the Bledow et al. (2011). Sample items are “I feel strong and vigorous in working for [...] platform” and “I am enthusiastic about working for [...] platform” (α = 0.897).

#### 3.2.5 Emotional stability

Emotional stability was measured with a 10-item scale developed by Li and Ahlstrom (2016). Sample items are “Can you recover from unhappiness quickly and not be influenced by it?” and “Can you recover from negative emotions quickly?” (α = 0.933).

#### 3.2.6 Perceived organizational support (POS)

POS was measured with a 6-item scale from Eisenberger et al. (1986). Sample items are “This organization cares about my wellbeing” and “This organization shows very little concern for me (reverse-coded)” (α = 0.801).

#### 3.2.7 Control variables

In line with previous study (Kundi et al., 2022), we controlled for gender, age, education level, and organizational tenure.

## 4 Results

### 4.1 Descriptive statistics

Table 1 presents the central tendencies, variability, and correlations among the variables of this study.

**Table 1:** Correlations and descriptive analysis.

**Variables**	**1**	**2**	**3**	**4**	**5**	**6**	**7**	**8**	**9**	**10**
1.Gender										
2. Age	−0.015									
3. Education level	−0.011	−0.032								
4. Tenure	0.093	0.142^*^	0.138							
5. AM	0.107	−0.019	−0.090	−0.025						
6. TA	−0.029	−0.264^***^	−0.146^*^	−0.201^**^	0.190^**^					
7. CA	−0.030	0.097	0.025	0.119^*^	0.187^**^	−0.184^**^				
8. WE	0.034	0.122	0.026	0.016	−0.041	0.316^***^	−0.256^***^			
9. ES	0.088	0.147^*^	0.081	0.085	−0.157^**^	−0.066	−0.027	0.126^*^		
9. POS	0.088	−0.120^*^	−0.118^*^	−0.186^**^	0.004	0.226^***^	−0.060	0.067	0.076	
Mean	1.130	24.30	1.811	1.37	3.057	2.972	2.289	3.691	3.125	2.755
SD	0.337	1.025	0.752	2.100	0.924	0.858	0.667	0.890	1.061	0.794

*N* = 292; AM, algorithmic management; TA, threat appraisal; CA, challenge appraisal; WE, work engagement; ES, emotional stability; POS, perceived organizational support; SD, standard deviation.

* *p* < 0.05,

** *p* < 0.01,

*** *p* < 0.001.

### 4.2 Measurement model analysis

Confirmatory factor analysis (CFA) was employed to validate the distinctiveness of the six key constructs in this research. As shown in Table 2, the proposed six-factor model demonstrated statistical significance in comparison to several alternative models (χ^2^ = 1038.465, *df* = 725, CFI = 0.947, TLI = 0.943, RMSEA = 0.044, SRMR = 0.045), thereby lending empirical evidence to the distinctive nature of the variables examined in our study. Furthermore, recognizing that all the data were self-reported by the delivery workers, we performed a CFA version of Harman's single-factor test to assess potential common method bias. The findings indicated that the single-factor model showed a severely inadequate fit to the data (χ^2^ = 5086.310, *df* = 740, CFI = 0.261, TLI = 0.221, RMSEA = 0.146, SRMR = 0.178), which sufficiently confirms that common method variance does not pose a significant issue.

**Table 2:** Confirmatory factor analysis.

**Model**	**χ^2^**	***Df***	***Δχ* ^2^**	**CFI**	**TLI**	**RMSEA**	**SRMR**
Six-factor Model	1038.465	725		0.947	0.943	0.044	0.045
Five-factor Model A	2790.839	730	1752.374^***^	0.650	0.626	0.102	0.131
Five-factor Model B	1492.045	730	453.58^***^	0.870	0.862	0.064	0.074
Five-factor Model C	1408.569	730	370.104^***^	0.885	0.877	0.061	0.069
One-factor model	5086.310	740	4047.845^***^	0.261	0.221	0.146	0.178

*** *p* < 0.001;

Six-factor Model: Algorithmic management, threat appraisal, challenge appraisal,emotional stability and POS; Five-factor model A: Algorithmic management and emotional stability were combined into one factor; Five-factor model B: Algorithmic management and POS were combined into one factor; Five-factor model C: threat appraisal and challenge appraisal were combined into one factor; One-factor model: Algorithmic management, threat appraisal, challenge appraisal,emotional stability and POS were combined into one factor.

### 4.3 Hypotheses testing

#### 4.3.1 Mediating effect testing

**Figure 2:**
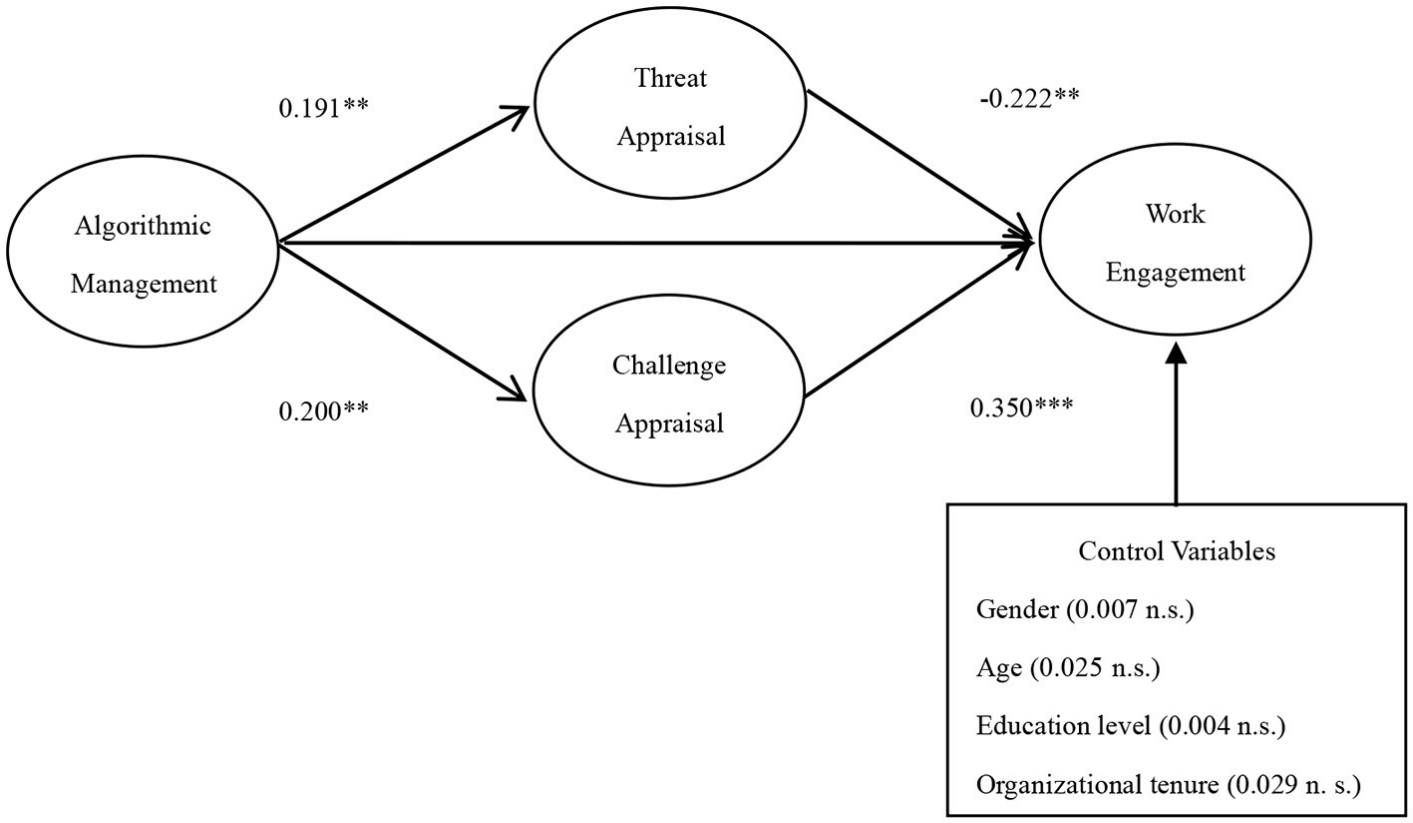
The results of structural model. *p < 0.05, **p < 0.01, ***p < 0.001.

Structural equation modeling (SEM) was performed with the assistance of MPLUS (7.4 edition) to test the hypotheses pertaining to mediation, and the result showed a fair fit to the data (χ^2^ = 400.231, *df* = 247, CFI = 0.955, TLI = 0.950, RMSEA = 0.054, SRMR = 0.055). The results of the structural model was shown in Figure 2. For the four control variables, none of them significantly influenced work engagement. PROCESS in SPSS (23 edition) was used to test the hypotheses pertaining to moderation and moderated mediation.

Hypothesis 1 proposed that algorithmic management has a negative indirect impact on work engagement through threat appraisal. As shown in Figure 2, the indirect effect of algorithmic management on work engagement, via threat appraisal, was negative and significant (β = −0.042, 95% CI = [−0.075, −0.010]). Therefore, Hypothesis 1 was supported. Hypothesis 2 suggested that algorithmic management has a positive indirect impact on work engagement through challenge appraisal. As shown in Figure 2, the indirect effect of algorithmic management on work engagement, via challenge appraisal, was positive and significant (β = 0.070, 95% CI = [0.026, 0.114]), supporting Hypothesis 2.

#### 4.3.2 Moderating effect testing

**Figure 3:**
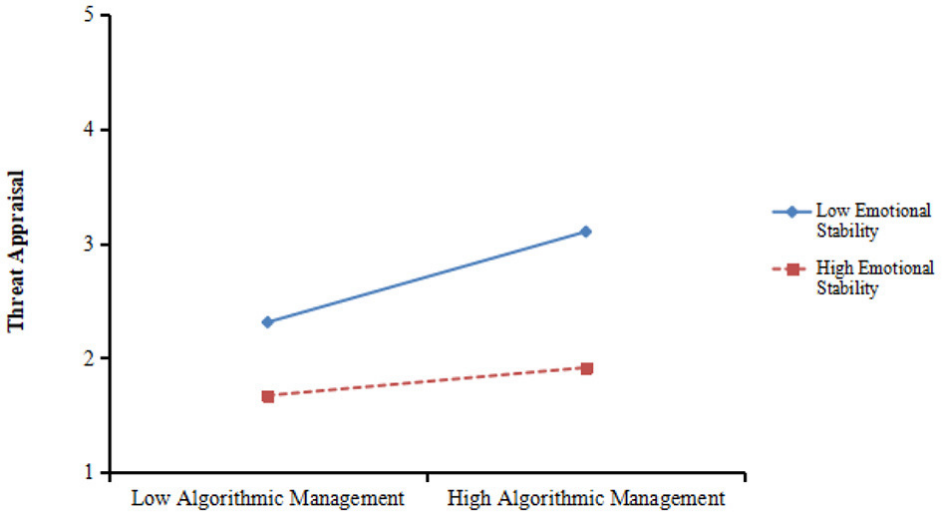
Interaction of emotional stability and algorithmic management on threat appraisal.

Then we tested the moderating effects of emotional stability and POS in the relationship between algorithmic management and work engagement by employing PROCESS in SPSS (23 edition). As shown in Table 3, the interaction between algorithmic management and emotional stability was significantly and negatively related to threat appraisal (β = −0.137, *p* < 0.01, model 1). To clearly interpret the interaction effects, the simple slope test for the interaction effects was plotted (see Figure 3), and the result revealed that the linkage between algorithmic management and threat appraisal was significant when emotional stability was low (−1SD, β = 0.396, *p* < 0.001) rather than high (+1SD, β = 0.122, *ns*). Thus, Hypothesis 3a was supported.

**Table 3:** The test of moderating effects.

**Variables**	**Threat appraisal**		**Challenge appraisal**	
	**Model 1**	**Model 2**	**Model 3**	**Model 4**
Algorithmic management	0.259^**^ (0.153)	0.470^***^ (0.131)	0.568^***^ (0.115)	0.186^**^ (0.169)
Emotional stability	−0.457^**^ (0.150)		0.431^***^ (0.115)	
POS		−0.361^**^ (0.148)		0.164^**^ (0.191)
Algorithmic management × Emotional stability	−0.137^**^ (0.035)		0.136^**^ (0.045)	
Algorithmic management × POS		−0.219^**^ (0.045)		0.131^*^ (0.058)

* *p* < 0.05,

** *p* < 0.01,

*** *p* < 0.001; *N* = 292.

**Figure 4:**
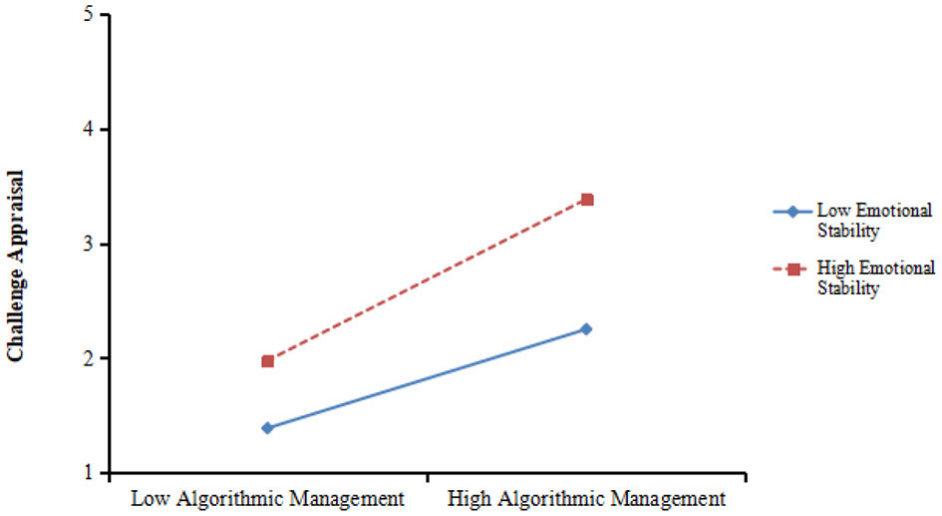
Interaction of emotional stability and algorithmic management on challenge appraisal.

In addition, as shown in Table 3, the interaction term between algorithmic management and emotional stability was significantly and positively influenced challenge appraisal (β = 0.136, *p* < 0.01, model 3). The plot is shown in Figure 4. And the result of simple slope analysis demonstrated that the linkage between algorithmic management and challenge appraisal was significant when emotional stability was high (+1SD, β = 0.704, *p* < 0.001) rather than low (−1SD, β = 0.182, *ns*). Therefore, Hypothesis 3b was supported.

**Figure 5:**
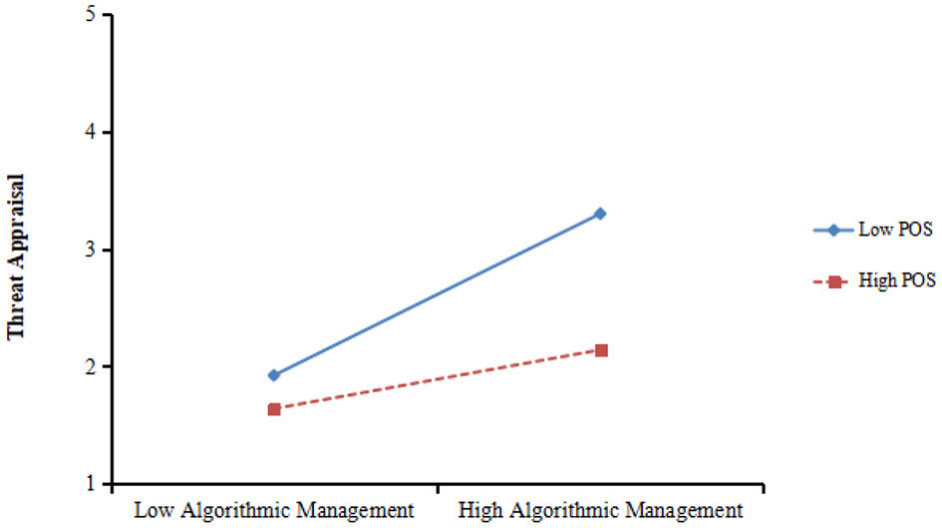
Interaction of POS and algorithmic management on threat appraisal.

Hypothesis 4a stated that the positive effect of algorithmic management on threat appraisal will be stronger when POS was lower than higher. As shown in Table 3, the interaction term between algorithmic management and POS was significantly and negatively related to threat appraisal (β = −0.219, *p* < 0.01, model 2). The plot is shown in Figure 5. And the result revealed that the linkage between algorithmic management and threat appraisal was significant when POS was lower (−1SD, β = 0.689, *p* < 0.001) rather than higher (+1SD, β = 0.125, *ns*). Thus, Hypothesis 4a was supported by data.

**Figure 6:**
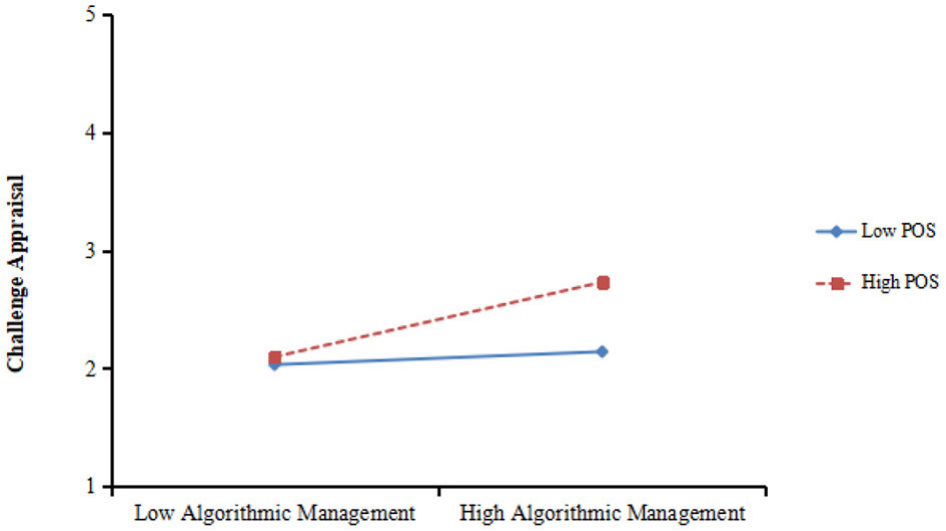
Interaction of POS and algorithmic management on challenge appraisal.

Hypothesis 4b suggested that the positive effect of algorithmic management on challenge appraisal will be stronger when POS was higher than lower. As can be seen in Table 3, the interaction term between algorithmic management and POS was significantly and positively influenced challenge appraisal (β = 0.131, *p* < 0.05, model 4). The plot is shown in Figure 6. And the result of simple slope analysis demonstrated that the linkage between algorithmic management and challenge appraisal was significant when emotional stability was higher(+1SD, β = 0.317, *p* < 0.001) rather than lower (−1SD, β = 0.055, *ns*). Therefore, Hypothesis 4b was supported.

#### 4.3.3 Moderated mediation effects testing

We tested the moderated mediation effects by employing PROCESS in SPSS (23 edition). As illustrated in Table 4, the indirect effect of algorithmic management on work engagement via threat appraisal was stronger when emotional stability was lower (β = −0.072, 95% CI = [−0.138, −0.028]) rather than higher (β = −0.001, 95% CI = [−0.033, 0.047]). The index of moderated mediation was significant (Index = 0.034, 95% CI = [0.009, 0.077]). Therefore, Hypotheses 5a was supported.

**Table 4:** Results of conditional indirect relationships.

**Conditional indirect relationships**	**B**	**SE**	**95% bias-corrected CI**
**Algorithmic management** → **Threat appraisal** → **Work engagement**			
Emotional stability (−1SD)	−0.072	0.278	[−0.138, −0.028]
Emotional stability (*M*)	−0.035	0.159	[−0.078, −0.011]
Emotional stability (+1SD)	−0.001	0.196	[−0.033, 0.047]
**Index of moderated mediation**	0.034	0.169	[0.009,0.077]
POS (−1SD)	−0.079	0.028	[−0.142, −0.030]
POS (*M*)	−0.034	0.015	[−0.073, −0.011]
POS (+1SD)	−0.009	0.015	[−0.151, 0.048]
**Index of moderated mediation**	0.055	0.022	[0.021, 0.110]
**Algorithmic management** → **Challenge appraisal engagement** → **Work**			
Emotional stability (−1SD)	0.050	0.021	[−0.039,0.055]
Emotional stability (*M*)	0.069	0.023	[0.017,0.101]
Emotional stability(+1SD)	0.095	0.032	[0.041,0.171]
**Index of moderated mediation**	0.041	0.017	[0.012,0.082]
POS (−1SD)	0.021	0.029	[−0.32,0.085]
POS (*M*)	0.053	0.022	[0.106,0.180]
POS(+1SD)	0.084	0.028	[0.150,0.379]
**Index of moderated mediation**	0.039	0.023	[0.002,0.092]

Also, as shown in Table 4, the indirect effect of algorithmic management on work engagement via challenge appraisal was stronger when emotional stability was higher (β = 0.095, 95% CI = [0.041, 0.171]) rather than lower (β = 0.050, 95% CI = [−0.039, 0.055]). The index of moderated mediation was significant (Index = 0.041, 95% CI = [0.012, 0.082]). Hence, Hypothesis 5b were supported.

Hypothesis 6a proposed that the indirect effects of algorithmic management on work engagement via threat appraisal will be stronger when POS is lower than higher. The result of Table 4 suggested that the indirect effect of algorithmic management on work engagement via threat appraisal was stronger when POS was lower (β = −0.079, 95% CI = [−0.142, −0.030]) rather than higher (β = −0.009, 95% CI = [−0.151, 0.048]). The index of moderated mediation was significant (Index = 0.055, 95% CI = [0.021, 0.110]). Therefore, Hypothesis 6a was supported.

Hypothesis 6b proposed that the indirect effects of algorithmic management on work engagement via challenge appraisal will be stronger when POS is higher rather than lower. As shown in Table 4, the indirect effect of algorithmic management on work engagement via challenge appraisal was stronger when POS was higher (β = 0.084, 95% CI = [0.150, 0.379]) rather than lower (β = 0.021, 95% CI = [−0.32, 0.085]). The index of moderated mediation was significant (Index = 0.039, 95% CI = [0.002, 0.092]). Hence, Hypothesis 6b was supported.

## 5 Discussion

Our research aimed to uncover the correlation between algorithmic management and employee engagement. Drawing on the transactional stress theory (Lazarus and Folkman, 1984), we hypothesized that algorithmic management would yield both negative and positive effects on delivery workers' work engagement. Appraising algorithmic management as a threat leads to stressful experiences and diminishes individual work engagement. On the contrary, appraising algorithmic management as a challenge can enhance employee work engagement. Moreover, the result also demonstrated that personal and job resources (i.e., emotional stability and POS, respectively) play a critical role in shaping how delivery workers perceive algorithmic management. The findings largely corroborate the theoretical predictions, yielding significant implications for both algorithmic management research and work engagement literature. In particular, when employees can obtain important items, energy, and social resources from their organizational context (Sarwar et al., 2020), they are more likely to appraise this pressure as a challenge rather than a threat and conduct better behavior such as work engagement, learning behavior, task performance, work proactivity, employee resilience (Chen et al., 2024; Ma et al., 2021; Shao et al., 2025). Moreover, POS moderated both the mediating effect of challenge appraisals in transmitting the positive effect of algorithmic management to work engagement and the mediating effect of threat appraisals in transmitting the negative effect of algorithmic management to work engagement. The conclusion of this study aligns with existing research, which demonstrates the buffering effects of POS on the relationship of stress and employee attitudes (Andra et al., 2022). In addition, emotional stability was an influential personal resource that impacted how one appraises pressure. This conclusion is also supported by prior studies. For instance, research by Kundi et al. (2022) demonstrated that emotional stability served as a moderator, indicating that employees perceived performance pressure as a challenge (vs. a threat), thereby boosting work engagement. High emotional stability workers were more likely to appraise the pressure as low threatening.

### 5.1 Theoretical implications

This study holds various implications for theoretical frameworks. First, moving beyond simplistic categorizations, this research conceptualizes algorithmic management as an integrated system that simultaneously generates enabling and constraining effects on gig workers' behavioral and attitudinal outcomes, as evidenced in China's food delivery industry. The current literature on algorithmic management remains trapped in a simplistic duality: while the field has attracted increasing research attention, scholars have largely failed to move beyond the reductive 'good-or-bad' paradigm in examining algorithmic management's organizational impacts. Previous literature on the impact of algorithmic management on workers could be divided into two main schools, namely, the positive school and the negative school, which respectively holds the perspectives that algorithmic management have a positive effect (Pei et al., 2021; Rosenblat and Stark, 2016; Kellogg et al., 2020) or a negative effect (Lei, 2021; Ashford et al., 2018; Shevchuk et al., 2019) on employees. Extending this perspective, our research advances a more nuanced understanding of algorithmic management by simultaneously examining the enabling and burdening effects of algorithmic management on the food delivery employees' work engagement, which not only provides a promising perspective for explaining the inconsistent findings in previous research but also facilitates a dialectical perspective of the comprehensive impact of algorithmic management.

Second, building on the TTS theory, we unveil the underlying mechanism of how algorithmic management might support or undermine work engagement. Prior work has explained the underlying underpinning of algorithmic management from the perspective of job demands—resources (JD-R) model (Li et al., 2025), work design theory (Parent-Rocheleau and Parker, 2022), psychological ownership theory (Liu et al., 2025). We add to the current literature by offering a stress appraisal perspective to understand the mechanisms linking algorithmic management and work engagement. Our work confirmed the mediating roles of challenge and threat appraisals in the relationship between algorithmic management and work engagement of delivery workers. Specifically, when employees perceive algorithmic management as a developmental opportunity, it activates a challenge appraisal pathway, thereby significantly enhancing work engagement. Conversely, when interpreted as an occupational threat, it triggers a threat appraisal pathway, ultimately undermining work engagement.

Third, our study also benefits existing literature on POS by investigating the moderating role of POS targeted at two types of appraisals (i.e., Challenge appraisal and threat appraisal). We found that POS enhances the effects of algorithmic management on challenge appraisal, whereas weakens the effects of algorithmic management on threat appraisal. The result demonstrated that the consequences of algorithmic management can be mixed unless workers exhibit high levels of POS as POS provides an optimistic perspective through which workers may appraise algorithmic management as challenging rather than threatening. Sarwar et al. (2020) argued that POS represents a key job resource to effectively cope with stress. Previous studies have suggested that social support has a cushioning effect in the relation between stressors and stress reactions (Kaufmann and Beehr, 1986; Scott et al., 2014; Viswesvaran et al., 1999).

Moreover, our study also contributes to the emotional stability literature by exploring how a single stressor (i.e., algorithmic management) could trigger different reaction (i.e., Challenge appraisal and threat appraisal), ultimately affecting the work engagement of workers. Based on cognitive appraisal theory of stress, we tested the moderating role of emotional stability on the relationship between algorithmic management and work engagement. Our study revealed that workers with high emotional stability are more likely to perceive the algorithmic management style as challenging rather than threatening, which promoting work engagement. Workers with high levels of emotional stability are more likely to remain calm and adopt effective coping strategies in stressful situations (Kundi et al., 2022). As such, we argued that emotional stability could be both functional (when high) and dysfunctional (when low) in perceiving algorithmic management. In doing so, our study enriches the knowledge of the consequences of algorithmic management and emotional stability by identifying workers who are more easily affected by the pressure due to algorithmic management style.

### 5.2 Practical implications

Our study also has several practical implications. First, our finding indicated that algorithmic management style of online labor platforms could be a double-edged sword, producing both positive and negative consequences. When algorithmic management is appraised as a threat, it may lead to dysfunctional behavior. However, when algorithmic management is seen as a challenge, it may strengthen functional behavior. Accordingly, managers of online food delivery platforms should be aware of how their workers appraise algorithmic management style. The platform manager can specifically understand the riders' views on algorithmic management through the algorithm hearing system, so as to implement improved algorithms and make the algorithm more user-friendly. Furthermore, platform managers can implement gamified training programs to regularly conduct challenge appraisal exercises for riders, guiding them to perceive algorithmic management as a positive challenge.

Second, our study suggested that the algorithmic management approach of online food delivery platforms could be beneficial to work engagement. Therefore, if managers want to motivate employees to engage in work, they should consider how they appraise this approach. When workers appraise this management approach as threatening or harmful, it may reduce their work engagement. However, if algorithmic management was appraised as challenging or helpful, it may motivate them to increase their work engagement. It should be emphasized that prolonged exposure to omnidirectional algorithmic surveillance may incur detrimental effects on workers. Accordingly, food delivery platform managers should: implement comprehensive task-support systems (e.g., manual review mechanisms for exceptional cases) to prevent unjust algorithmic penalties; adopt participatory governance models, such as allocating rider representation seats on algorithmic oversight committees; establish quantitative fairness metrics (e.g., disparity testing scores) to enhance algorithmic transparency and mitigate adverse consequences.

Third, our study found that delivery workers with high emotional stability are more sensitive to the challenging aspects of algorithmic management while seemingly ignoring the threatening aspects of algorithmic management, and they were found to have greater engagement behavior at work. In the case of low emotional stability, they are more likely to appraise algorithmic management as a threat, thereby lowering their work engagement. Therefore, managers might pay more attention to the emotional stability of delivery workers. For example, during the selection and recruitment process, human resource managers could ask candidates to fill out relevant scales of emotional stability and prioritize hiring only those with high emotional stability, as they are more likely to appraise algorithmic management from a positive perspective (i.e., Challenge appraisal). Furthermore, for the employed personnel, interventions such as training, counseling and mentoring should be arranged to improve the emotional stability of workers.

Finally, our study revealed that increasing POS may help enhance the positive effect of algorithmic management on challenge appraisal and weaken the positive effect of algorithmic management on threat appraisal. In the case of high POS, they are more likely to appraise algorithmic management as a threat, thereby increasing their work engagement. One way to help delivery workers to form challenge appraisal toward algorithmic management may be to increase their belief they are cared for and supported by their organization in the work process controlled by algorithm technology. In other words, food delivery platforms should attempt to increase workers' perceptions of the amount of support they receive from the platforms, may be through management policies containing improving rewards, building a warm working environment and treating everyone equally (Rhoades and Eisenberger, 2002).

### 5.3 Limitations and future research direction

Our research inevitably has some limitations. First, concerns about common method bias should be mentioned since all the variables of our study were collected by self-reported scales. The CMV issues in our surveys were addressed by (1) employing two-wave data collection procedure, (2) using MPLUS to conduct CFA marker variable analyses which demonstrated that CMV may not be a threat to our study and (3) included a moderator variable in our theoretical framework which prior studies have suggested cannot be inflated by CMV (Podsakoff et al., 2012). Our study found that the interaction effect of psychological capital and the indirect effect of algorithmic management on work engagement were both significant, suggesting that CMV unlike to be a problem. However, future research should try to enhance causal relationships and reduce the CMV by adopting alternative research designs, such as experiments or longitudinal studies.

Second, as our study was conducted among food delivery workers in China, the generalizability of our results is limited. Future research may benefit from examining our theoretical model within other industries or even from different countries and cultures. Finally, our study has proved the moderating roles of emotional stability and POS on weakening the negative consequence of algorithmic management on threat appraisal. Future research could explore the moderating role of other personality trait (e.g., regulatory focus) and other organizational context (e.g., transformation leadership) which may increase or reduce the salience of stressors.

## Data Availability

The original contributions presented in the study are included in the article/supplementary material, further inquiries can be directed to the corresponding authors.
